# Supplementary material for: Emotional impact according to the way cancer patients are conducted to the surgical center: A randomized clinical trial comparing ambulation to the stretcher
Source: PLoS One. 2025 Apr 22;20(4):e0320856. doi: 10.1371/journal.pone.0320856 (PMC12013869; doi:10.1371/journal.pone.0320856)
Supplement: S1 Protocol — (DOCX) [file pone.0320856.s001.docx]

**BARRETOS CANCER HOSPITAL**

**"Impact on Anxiety Levels According to the way of Transporting Cancer Patients to the Surgical Center: A Randomized Clinical Trial Comparing Transport via Stretcher and Ambulation."**

**Principal Investigator:** Prof. Dr. Ricardo dos Reis

**Other Researchers:** Gabriela da Silva Oliveira, Ana Carolina de Matos Magalhães, Marcelo A. Vieira, Carlos Andrade, Audrey Tsunoda, Carlos Eduardo Paiva.

**BARRETOS-SP**

**2020**

**SUMMARY**

**Introduction:**Cancer is an extremely aggressive disease, and anxiety and depression are consequences that some patients may develop, from the time of diagnosis, continuing throughout the course of treatment. One of the measures to control cancer is surgery to remove the tumor. The surgical procedure is often a difficult experience for patients and their families, and patients in the preoperative period are often encountered with psychological symptoms of anxiety and depression. Studies show that patients welcomed by family members in the preoperative period significantly reduce the symptoms of anxiety, fear and panic compared to those who were only accompanied by health professionals. **Goals:**To analyze the emotional repercussion of patients diagnosed with cancer classified as ECOG PS 0 and 1 according to the type of transport to the operating room. **Methodology:**Randomized clinical trial, with prospective collection, which will take place at the Barretos Cancer Hospital. For the sample calculation,considering a α error of 0.05 and a power of 1 – β of 0.80, a sample size of 176 patients was reached, with 88 patients allocated to the stretcher group, and 88 patients allocated to the ambulation group.**Expected results:**It is expected to find some emotional change in the degree of anxiety and satisfaction of patients according to the modification of their type of transport to the operating room, and it is possible to modify the hospital routine related to the way these patients are conducted.

**Keywords:**Anxiety, depression, operating room, ambulation, preoperative.

**1. INTRODUCTION**

As described by the World Health Organization (WHO), it is indisputable that cancer is a public health problem, especially among developing countries, where it is expected that in the coming decades the impact of cancer on the population will correspond to 80% of the more than 20 million new cases estimated for 2025 ([1](#_ENREF_1)).

Cancer is an extremely aggressive disease, and the science of the disease has a great impact on the patient's life, and can cause physical and emotional changes. Anxiety and depression are consequences that some patients can develop, from the time of diagnosis, continuing throughout the course of treatment ([2](#_ENREF_2), [3](#_ENREF_3)).

There are several types of cancer treatment, including surgery, chemotherapy, radiotherapy, hormone therapy, immunotherapy and rehabilitation. And some types of treatments bring numerous side effects related to the fact that they do not exclusively reach cancer cells, among them the following stand out: nausea, vomiting, alopecia and burns that weaken the patient and shake his emotional state, which corroborates the hopelessness and suffering of the patient diagnosed with cancer ([4](#_ENREF_4)).

One of the measures to control cancer is surgery to remove the tumor, however, although this is often possible, some surgical practices can have serious physical consequences, such as surgical procedures with mutilation, and the fear of surgical intervention in some cancer patients is so great that pathological anxiety or distorted thoughts result in refusal of treatment in more than 5% of cases ([5](#_ENREF_5), [6](#_ENREF_6)).

Patients in the preoperative period are often found with psychological symptoms of anxiety and depression, and the ideal would be that there are no concerns other than those caused by the disease itself. However, anticipation of pain, separation from family, loss of independence, fear of disability, in addition to fear of the procedure itself and death, end up being factors that frequently trigger these symptoms during this period ([7](#_ENREF_7)).

In a previous study, it was observed that preoperative anxiety was present in about 80% of adult patients who were waiting for some type of surgery. In view of this, anxiety deserves due attention from the health team, as it can influence the patient's response to treatment and cause negative effects on their postoperative recovery. In addition, anxiety causes physiological changes, such as tachycardia and arterial hypertension, with a consequent increase in oxygen consumption and worsening of the evolution of the disease ([8](#_ENREF_8)).

The surgical procedure is often a difficult experience for patients and their respective families, and requires a series of affective mobilizations to deal with the anxiety and stress of the situation. Any surgical act is considered a critical situation capable of arousing individual behaviors, influenced by multiple physical and emotional factors ([9](#_ENREF_9)).

One of the strategies used to reduce anxiety and depression levels during the preoperative period is to provide information about health conditions, procedures performed and the participation of the family member at this time ([9](#_ENREF_9)).

Studies show that patients welcomed by family members in the preoperative period significantly reduce the symptoms of anxiety, fear and panic compared to those who were only accompanied by health professionals. The presence of the family member mainly reduces the feeling of anxiety in the preoperative period, characterized as a feeling of fear and apprehension of the unknown, contrary to what happens when there is a single presence of the nursing professional, increasing the anxiety rates in relation to patients who were not accompanied by their family members. Thus, it is possible to see the essential relationship between the presence of family members and the reduction of anxiety in such a critical period, which is the preoperative period ([8](#_ENREF_8)).

Another important point is that in the literature it is very common to find articles that define the importance of intra-hospital transport for diagnostic or therapeutic purposes of critical patients, including those who have some type of cancer being treated in the Intensive Care Unit (ICU). The transfer of these patients depends directly on the planning and organized action of the multidisciplinary team, as well as the choice of indispensable equipment, which is necessary because critically ill patients are more susceptible to physiological changes, which can cause complications along the way. However, little has been studied about transportation to the operating room when the patient is in a non-critical state ([8](#_ENREF_8), [10](#_ENREF_10), [11](#_ENREF_11)).

Kojima e*t al.* ([12](#_ENREF_12)) conducted a study between October of 1998 and September of 2000 with patients aged 15 to 80 years who were to undergo some type of surgery, but the study did not specify the previous diseases or the reason for the surgery, giving due importance only to the way of transportation to the operating room: one through the use of a stretcher and the other through ambulation, also analyzing the patients' degree of awareness of the medications used, reaching the following result: patients who walk feel calmer in the preoperative period. The use of stretchers makes them more anxious, distressed and even inferior when talking to other people since they remain lying down and draw more attention wherever they go. All of this reflects the stress caused by this type of transport, and it is understood that ambulation reduces the level of anxiety caused by this type of transport. However, in this study, the diseases such as cancer, the degree of limitation of the patients, or the classification in the PS score of the patients submitted to the surgical procedure are not specified. Another important point is the wide spectrum of age range of the patients analyzed by the study, since it encompasses from adolescents to the elderly, generating many variables for a truthful analysis of the results.

1. **JUSTIFICATION**

Cancer patients present emotional changes from the moment of their diagnosis and throughout the treatment, for this reason, the study wants to observe if there is any change in the emotional responses, the degree of anxiety and the satisfaction of adult cancer patients classified as PS 0 and 1 according to the change in the type of transport to the operating room, replacing the use of stretchers (traditional model) with ambulation. In addition, there is a deficit of studies published in the literature on this topic, especially in cancer patients, and according to the final findings, it can change the daily practice in relation to the way these patients are taken to the operating room, reducing anxiety levels and thus resulting in a better recovery of the patient.

- 1. **Factor under Study**

Way of driving to the operating room (stretcher versus ambulation)

- 1. **Primary outcome**

Emotional impact according to the way they were driven to the operating room.

1. **OBJECTIVES**
   1. **Primary objective**

**3.1.1** Determine the anxiety levels of the patient diagnosed with cancer according to the type of transport to the operating room.**.**

**3.2 Secondary objectives**

**3.2.1** To analyze whether the type of transport of the patient classified as PS 0 and 1 has an influence on the degree of depression.

**3.2.2** To analyze whether the type of patient transport classified as PS 0 and 1 has an influence on the degree of patient satisfaction.

**3.2.3**Analyze the patient's anxiety according to the type of transport, according to gender.

**3.2.4** Analyze the patient's anxiety according to the type of transport, according to the socioeconomic level.

1. **METHODOLOGY**

**4.1 Design**

This is a randomized clinical trial, with prospective collection, to analyze the emotional repercussion of patients diagnosed with cancer classified as ECOG PS 0 and 1 according to the type of transport to the operating room.

**4.2 Sample**

To select these patients, we will use the fall risk assessment scale adopted in the hospital routine for patients over 60 years of age, together with the *Eastern Cooperative Oncology Group (ECOG PS)*scale (Table 1), with only patients being classified as no risk or with minimal risk,*,* according to the fall risk scale (Table 2), and patients with SP 0 and 1.

**Table 1 -ECOG Performance Status** (Grupo de Oncologia*Eastern Cooperative, Robert L. Comis, MD.)*

| Degree | **ECOG PERFORMANCE STATUS** |
| --- | --- |
| 0 | Fully active, able to develop all your performance, without any restrictions. |
| 1 | Restrictions on strenuous activities, while maintaining normality in their daily practice. |
| 2 | He still retains his capacity for self-care, but he is unable to perform any work activity, and if he does, it is in less than 50% of waking hours. |
| 3 | Limited capacity for self-care; confined to a bed or chair for 50% of waking time. |
| 4 | Completely disabled, unable to exercise self-care, totally confined to a bed or chair. |
| 5 | Death |

**Table 2: -**SCALE FOR ASSESSING THE RISK OF FALLING (Adapted from Jonhs Hopkins 2007)

| Zero = No Risk 1-5 = Low Risk 6-13 = Moderate Risk > 13 = High Risk | |
| --- | --- |
| **Risk Factor** | **Punctuation** |
| 1. **Age**(select option 1) |  |
| •         60 – 69 years old | 1 |
| •         70 – 79 years old | 2 |
| •         Greater than or equal to 80 years old | 3 |
| 2. **Fall history** |  |
| •         Drop in the last 6 months before admission | 5 |
| 3. **Eliminations**(select option 1): |  |
| •         Incontinence | 2 |
| •         Urgency or change in frequency | 2 |
| •         Urgency/change in frequency and incontinence | 4 |
| 4. **Medications** |  |
| •         Use of 1 of the options | 3 |
| •         Use of 2 or more options | 5 |
| •         Underwent sedative procedures within the last 24 hours | 7 |
| 5. **Use of Equipment:**any equipment used by the patient, such as catheters, venipuncture, among others (select 1 option) |  |
| •         Presence of 1 device | 1 |
| •         Presence of 2 pieces of equipment | 2 |
| •         Presence of 3 or more devices | 3 |
| 6. **Mobility**(multiple selection allowed) |  |
| •         Needs assistance or supervision for movement, transfer or ambulation | 2 |
| •         Unstable gear | 2 |
| •         Visual and/or hearing impairment that affects movement | 2 |
| 7. **Cognitive**(multiple selection allowed): |  |
| •         Altered consciousness related to the environment | 1 |
| •         Impulsive | 2 |
| •         Lack of understanding of their physical and cognitive limitations | 4 |
| 8. **Special conditions:**According to the list: | No / Yes |

Health conditions and presence of chronic diseases – must be linked to item 8 of the scale:

Previous stroke, postural hypotension, dizziness, low body mass index, anemia, insomnia, incontinence or urinary urgency, arthritis, osteoporosis, metabolic alterations (e.g., hypoglycemia).

**Table 3 -**Medications related to the use of mean item 4.

| Atenolol | Dimenhydrinate | Regular Human Insulin | Nalbuphine | Sultamicilin |
| --- | --- | --- | --- | --- |
| Atracurium | Dipyridamole | NPH insulin | Naloxone | Succinylcholine |
| Atropine | Dipyrone | Potassium Iodide | Naproxen | Tracolimos |
| Methyl Blue | Dobutamine | Iodixanol (Visipaque Leaflet) | Neostigmine | Tacrolimus Topical |
| Benzydamine | Dopamine | Ioversol | Nifedipine | Theicoplanin |
| Betamethasone | Droperidol | Ipratropium | Nimesulide | Terbutaline |
| Bisacodyl | Epinephrine | Ipratropium + Fenoterol | Nitroglycerine | Thiabendazole |
| Bromopride | Ertapenem |  | Nitroprusside | Thiopental |
| Budesonide | Scopolamine | Dinitrate | Norfloxacin | Tramadol |
| Bupivacaine | Spironolactone | Itraconazole | Nortrititiline | Sodium Valproate |
| Captopril | Streptokinase | Ivermectin | Octreotide | Vancomycin |
| Carbamazepine | Ethylephrine | Lamivudine | Omeprazole | Warfarin |
| Carvedilol | Etomidate | Levofloxacin | Ondansetron | Vasopressin |
| Ceftriaxone | Phenazopyridine | Levomepromazine | Pancuronium | Verapamil |
| Ketamine | Phenytoin | Levothyroxine | Paracetamol | Voriconazole |
| Ketoprofen | Phenobarbital | Lindocaine | Paroxetine |  |
| Tromethamine ketorolac | Fenoterol | Lindocaine + Epinephrine | Pethidine |  |
| Cyclosporine | Fentalina | Lindocaine 25 mg/g + Prilocaine 25 mg/g 5g | Piperacillin |  |
| Cirpofloxacin | Phytomenadione (vitamin K) | Loperamide | Polymexin B |  |

**4.3 Sample calculation**

Based on the results found by Kojima e*t al.* ([12](#_ENREF_12)) A sample size was calculated to compare proportions. Considering a α error of 0.05 and a power of 1 – β of 0.80, a sample size of 176 patients was reached, with 88 patients allocated to the stretcher group, and 88 patients allocated to the ambulation group.

**4.4 Eligibility Criteria**

- Patients diagnosed cancer
- Age 18 years or older, up to 70 years old
- Both sexes
- Classified as PS score 0 or 1
- Patients who are rated Zero Risk or Low Risk by the falls risk assessment scale
- Indication for elective surgery

**4.5 Non-eligibility criteria**

- Patients who, moments before surgery, present an adverse event that may jeopardize walking to the operating room.
- Patients who have a physical disability and need help to walk.
- Patients who have an indication for a bed in the Intensive Care Unit (ICU)
- Patients who have previously diagnosed psychiatric disorders (severe depression, anxiety disorder, panic, among others).
- ECOG patients > 1.
- Patients who do not have a companion on the day of surgery.
- Patients using anxiolytic and antidepressant medications.

**4.6 Data Collection**

A research instrument will be made on the REDCap data platform. In it, we will collect socio-demographic data such as: date of admission, date of birth, weight, height, gender, ethnic group, current residence, contact telephone number, name of the companion and their degree of kinship, socioeconomic level, education. We will use the source document (medical record) for information such as: classification of malignant tumors (TNM), classification of performance status according to comorbidities, classification of the fall risk scale, which is performed at the time of the patient's hospitalization by the employee responsible for the same, medications in use, other surgeries, complementary exams confirming the presence of cancer, the type and location of the neoplasm, date of surgery, surgical size.

Patients will be identified as potential participants for the study at the time of their admission. Fulfilling all the eligibility criteria, it will be randomized randomly through the REDCap platform, and the orientation of which group the patient fell will be carried out, for the nursing professional (stretcher bearer) who will take him to the operating room.

Group 1 will be welcomed in their room by a nursing technician, a family member and the stretcher bearer who will take them lying on a stretcher to the operating room, accompanied by their family member, where they will be received by a nursing professional who will perform the standard procedures of the operating room. Group 2 will leave their room and go to the operating room walking with a family member and the stretcher bearer who will accompany them during the journey. Patients randomized to group 1 should be taken to the operating room in hospital clothes, while patients randomized to group 2 should be taken to the operating room with their own clothes. Upon entering the operating room, both patients in groups 1 and 2 will answer the questionnaire HAD scale – assessment of the level of anxiety and depression, easy to handle and quickly executed, which was developed to detect states of anxiety and depression in physically ill patients, who can answer it alone ([13](#_ENREF_13), [14](#_ENREF_14)) (APPENDIX 1), and the Questionnaire for the Evaluation of Satisfaction with Health Care in Surgery (Sati-Cir) (APPENDIX 2)), a questionnaire developed for the present study in order to evaluate patient satisfaction with the care received at the hospital, and to evaluate satisfaction with the way the patient was conducted to the operating room. For this questionnaire, a pilot test will be carried out with 20 patients who will have their way of conducting the operating room randomized randomly via RedCap, in order to analyze the patients' understanding of the questions asked.

After the application of both questionnaires, the Informed Consent Form (ICF) of the study will be applied. The application of the ICF will be carried out after the transport and application of the questionnaires in the operating room, so that there is no bias of the participant in the way he will answer the questionnaires, so as not to induce him to believe that being taken to the operating room walking is more comfortable and causes more satisfaction.

Both questionnaires will be used to evaluate the findings regarding emotional changes, degree of anxiety and satisfaction experienced by the patient at that moment, the questionnaires are self-administered or can be applied to the patient by members of the Researcher Support Center, and a scientific initiation student of the present study.

**4.7 Study location**

The present project will cover a prospective collection study of patients with elective surgical indication at the Barretos Cancer Hospital, a national reference center for the treatment of cancer patients.

**4.8 Randomização**

Randomization will be generated through the platform - REDCap - creating a random list of entry into the study, separating into group 1 - control (patients who will be transported to the operating room by the traditional method, on a stretcher) and group 2 - experimental (patients who will walk to the operating room), both groups accompanied by their families. In this way, all patients will have an equal chance of being included in both groups.

**4.9 Statistical analysis**

Initially, the data of our sample will be described through measures of central tendency (mean and median) and dispersion (standard deviation and quartiles) for all quantitative variables. For the qualitative variables, we will use tables containing the absolute and relative values of each category of variables. Subsequently, in order to observe the difference between the study groups (Stretcher x Walking) in relation to emotional repercussion, degree of satisfaction and anxiety, we will use statistical tests considering the significance of 0.05. If the variables to be listed have a Normal distribution, we will use the T-test, and if not, we will apply the Mann-Whitney test. For categorical variables, we will use the Chi-square or Fischer's exact tests, depending on the characteristics of the data. For such analyses, we will use the SPSS version 21 software, and the help of the Center for Epidemiology and Biostatistics (NEB) of the Barretos Cancer Hospital.

**5. ETHICAL ISSUES**

This project is ethically and methodologically in accordance with the Guidelines and Regulatory Standards for Research involving Human Beings (Resolution 466/12 of the National Health Council) and will be submitted to the Institutional Review Board (IRB) of the Barretos Cancer Hospital. Patients will sign an informed consent form to participate in the study (APPENDIX 3). Failure to sign the term will result in the patient's non-participation in the study, but will not interfere with the patient's treatment at the institution.

**5.1 Risks to the Participant**

There is a risk of breach of data confidentiality between the members of the study, but this will be avoided as much as possible, as well as the minimum risk of falls, since a specific group will be taken to the operating room walking. And to minimize these risks, we will use the fall risk scale used in the hospital's routine, not exposing any patient to complications. The patient will be accompanied by the stretcher bearer and his family member to the operating room, for possible support if he feels unwell during this journey.

**5.2 Benefit to the participant**

We considered finding a relationship between the way these patients were taken to the operating room and the degree of anxiety presented by the patient before the surgical procedure. It can change the routine of transporting patients to the operating room, since they are patients in good physical condition, and may add some psychological benefit, removing the idea of illness and incapacitation in the face of this simple journey.

**6. BUDGET**

The expenses of this study will be related to the printing of free and informed consent forms, database and questionnaire, therefore, only white sheets of A4 sulfite and the replacement of the black printer cartridge will be necessary, which will be financed with the researcher's private resources, which will be around R$200.00.

**7. SCHEDULE**

| **ACTIVITIES** | **2018** | **2019** | | **2020** | | | **2021** | |
| --- | --- | --- | --- | --- | --- | --- | --- | --- |
| **PERÍODO** | **P1** | **P1** | **P2** | **P1** | **P2** | **P3** | **P1** | **P2** |
| IRB | **X** |  |  |  |  |  |  |  |
| *Clinical Trials* Approval |  | **X** |  |  |  |  |  |  |
| Data Collection |  | **X** | **X** | **X** | **X** | **X** |  |  |
| Disciplines |  |  | **X** | **X** | **X** | **X** | **X** |  |
| Follow-up Boards |  |  |  | **X** |  | **X** |  | **X** |
| Statistical analysis |  |  |  |  |  |  | **X** | **X** |
| Discussion of Results |  |  |  |  |  |  | **X** | **X** |
| Thesis writing |  |  |  |  |  |  |  | **X** |
| Defense |  |  |  |  |  |  |  | **X** |

*P: 4 month period*

**REFERENCES**

1. Estimativa 2016 - Incidência de Câncer no Brasil Rio de Janeiro2015 [Available from: <http://www.inca.gov.br>.

2. Bailey RK, Geyen DJ, Scott-Gurnell K, Hipolito MM, Bailey TA, Beal JM. Understanding and treating depression among cancer patients. Int J Gynecol Cancer. 2005;15(2):203-8.

3. Jenkins PL, May VE, Hughes LE. Psychological morbidity associated with local recurrence of breast cancer. Int J Psychiatry Med. 1991;21(2):149-55.

4. Barbosa LNF SD, Amaral MX, Gonçalves AJ, Bruscato WL. Repercussões psicossociais em pacientes submetidos a laringectomia total por câncer de laringe: Um estudo clínico-qualitativo. Revista da Sociedade Brasileira de Psicologia Hospitalar. 2004(7(1)):45-58.

5. Bottomley A. Psychosocial problems in cancer care: a brief review of common problems. J Psychiatr Ment Health Nurs. 1997;4(5):323-31.

6. Braz DS, Ribas MM, Dedivitis RA, Nishimoto IN, Barros AP. Quality of life and depression in patients undergoing total and partial laryngectomy. Clinics (Sao Paulo). 2005;60(2):135-42.

7. Maranets I, Kain ZN. Preoperative anxiety and intraoperative anesthetic requirements. Anesth Analg. 1999;89(6):1346-51.

8. Assis CC, Lopes Jde L, Nogueira-Martins LA, de Barros AL. [Embracement and anxiety symptoms in patients before cardiac surgery]. Rev Bras Enferm. 2014;67(3):401-7.

9. Santos MAd, Rossi LA, Paiva L, Dantas RAS, Pompeo DA, Machado ECB. Medida da ansiedade e depressão em pacientes no pré-operatório de cirurgias eletivas. Rev eletrônica enferm. 2012;14(4):922-7.

10. Kelly CM, Shahrokni A. Moving beyond Karnofsky and ECOG Performance Status Assessments with New Technologies. J Oncol. 2016;2016:6186543.

11. Rossi L SM. Repercussões psicológicas do adoecimento e tratamento em mulheres acometidas pelo câncer de mama. Psicologia, Ciência e Profissão. 2003(23(4)):32-41.

12. Kojima Y, Ina H, Fujita T, Mitono H. Relieving anxiety by entering the operating room on foot. Can J Anaesth. 2002;49(8):885-6.

13. Magalhaes Filho LL, Segurado A, Marcolino JA, Mathias LA. [Impact of preanesthetic evaluation on anxiety and depression in cancer patients undergoing surgery.]. Rev Bras Anestesiol. 2006;56(2):126-36.

14. Marcolino JA, Mathias LA, Piccinini Filho L, Guaratini AA, Suzuki FM, Alli LA. Hospital Anxiety and Depression Scale: a study on the validation of the criteria and reliability on preoperative patients. Rev Bras Anestesiol. 2007;57(1):52-62.

**APPENDIX 1. Hospital Anxiety and Depression Scale (HADS)(**[**14**](#_ENREF_14)**).**

**Tick the box beside the reply that is closest to how you have been feeling in the past week.**

**Don’t take too long over you replies: your immediate is best.**

| **D** | **A** |  | **D** | **A** |  |
| --- | --- | --- | --- | --- | --- |
|  |  | **I feel tense or 'wound up':** |  |  | **I feel as if I am slowed down:** |
|  | 3 | Most of the time | 3 |  | Nearly all the time |
|  | 2 | A lot of the time | 2 |  | Very often |
|  | 1 | From time to time, occasionally | 1 |  | Sometimes |
|  | 0 | Not at all | 0 |  | Not at all |
|  |  |  |  |  |  |
|  |  | **I still enjoy the things I used to enjoy:** |  |  | **I get a sort of frightened feeling like 'butterflies' in the stomach:** |
| 0 |  | Definitely as much |  | 0 | Not at all |
| 1 |  | Not quite so much |  | 1 | Occasionally |
| 2 |  | Only a little |  | 2 | Quite Often |
| 3 |  | Hardly at all |  | 3 | Very Often |
|  |  |  |  |  |  |
|  |  | **I get a sort of frightened feeling as if something awful is about to**  **happen:** |  |  | **I have lost interest in my appearance:** |
|  | 3 | Very definitely and quite badly | 3 |  | Definitely |
|  | 2 | Yes, but not too badly | 2 |  | I don't take as much care as I should |
|  | 1 | A little, but it doesn't worry me | 1 |  | I may not take quite as much care |
|  | 0 | Not at all | 0 |  | I take just as much care as ever |
|  |  |  |  |  |  |
|  |  | **I can laugh and see the funny side**  **of things:** |  |  | **I feel restless as I have to be on the**  **move:** |
| 0 |  | As much as I always could |  | 3 | Very much indeed |
| 1 |  | Not quite so much now |  | 2 | Quite a lot |
| 2 |  | Definitely not so much now |  | 1 | Not very much |
| 3 |  | Not at all |  | 0 | Not at all |
|  |  | **Worrying thoughts go through my**  **mind:** |  |  | **I look forward with enjoyment to**  **things:** |
|  | 3 | A great deal of the time | 0 |  | As much as I ever did |
|  | 2 | A lot of the time | 1 |  | Rather less than I used to |
|  | 1 | From time to time, but not too often | 2 |  | Definitely less than I used to |
|  | 0 | Only occasionally | 3 |  | Hardly at all |
|  |  |  |  |  |  |
|  |  | **I feel cheerful:** |  |  | **I get sudden feelings of panic:** |
| 3 |  | Not at all |  | 3 | Very often indeed |
| 2 |  | Not often |  | 2 | Quite often |
| 1 |  | Sometimes |  | 1 | Not very often |
| 0 |  | Most of the time |  | 0 | Not at all |
|  |  |  |  |  |  |
|  |  | **I can sit at ease and feel relaxed:** |  |  | **I can enjoy a good book or radio or TV**  **program:** |
|  | 0 | Definitely | 0 |  | Often |
|  | 1 | Usually | 1 |  | Sometimes |
|  | 2 | Not Often | 2 |  | Not often |
|  | 3 | Not at all | 3 |  | Very seldom |

Please check you have answered all the questions Scoring:

Total score: Depression (D) Anxiety (A)

0-7 = Normal

8-10 = Borderline abnormal (borderline case)

11-21 = Abnormal (case)

**APPENDIX 2: Questionnaire for Assessment of Satisfaction with Health Care in Surgery (Sati-Cir).**

This questionnaire aims to assess your SATISFACTION with the health care received in this hospital, mainly in relation to issues related to surgery. It is composed of two parts: Part A contains 8 items with 5 response options, ranging from 1 (very satisfied) to 5 (very dissatisfied). In part B, two complementary questions assess your satisfaction with the care received, with qualitative answer options (with more detailed explanations).

**Part A**

|  | Very Satisfied | Satisfied | Indifferent | Dissatisfied | Very Dissatisfied |  |  |  |  |  |
| --- | --- | --- | --- | --- | --- | --- | --- | --- | --- | --- |
| 1. *How satisfied are you with clarifying doubts about your illness?* | 1 | 2 | 3 | 4 | 5 |  |  |  |  |  |
| 1. *How satisfied are you with your participation in decisions related to your treatment?* | 1 | 2 | 3 | 4 | 5 |  |  |  |  |  |
| 1. *How satisfied are you with the opportunities the team gives you to ask questions?* | 1 | 2 | 3 | 4 | 5 |  |  |  |  |  |
| 1. *How satisfied are you with the way you get information when you ask for it?* | 1 | 2 | 3 | 4 | 5 |  |  |  |  |  |
| 1. *How satisfied are you with the care provided by the nursing team to preserve your privacy?* | 1 | 2 | 3 | 4 | 5 |  |  |  |  |  |
| 1. *How satisfied are you with the time you waited until you were admitted?* | 1 | 2 | 3 | 4 | 5 |  |  |  |  |  |
| 1. *How satisfied are you with the time you waited until the surgery?* 2. *How were you taken to the surgical center:* | 1 | 2  stretcher bearer | 3 | 4  walking | 5 |  |  |  |  |  |
| 1. *How satisfied are you with the way you were taken to the surgical center?* 2. *How were you taken to the surgical center:* | 1 | 2  Pajama | 3 | 4  their own clothes | 5 | 1 | 2 | 3 | 4 | 5 |
| 1. *How satisfied are you with the clothing you wore when taken to the surgical center?* | 1 | 2 | 3 | 4 | 5 |  |  |  |  |  |

**Part B**

**Regarding the clothing you were taken to the surgical center, answer:**

Did you think that your dignity/privacy was preserved in relation to the clothing in which you were taken to the surgical center?

( ) Yes

( ) No, why:________________________________________________________________

**Regarding the way you were taken to the surgical center, answer:**

Did you think that your **autonomy** was preserved in relation to the way you were taken to the surgical center?

( ) Yes

( ) No, why:________________________________________________________________

If you could choose how to be taken to the surgery center, what would it be?

( ) stretcher bearer

( ) Wheelchair

( ) walking

**APPENDIX 3 the Informed Consent Form (ICF)**

**the Informed Consent Form (ICF)**

**Impact on Anxiety Levels According to the way of Transporting Cancer Patients to the Surgical Center: A Randomized Clinical Trial Comparing Transport via Stretcher and Ambulation.**

**Principal Investigator:** Prof. Dr. Ricardo dos Reis

**Other Researchers:** Gabriela da Silva Oliveira, Ana Carolina de Matos Magalhães, Marcelo A. Vieira, Carlos Andrade, Audrey Tsunoda, Carlos Eduardo Paiva.

**WHAT IS THIS DOCUMENT?**

You are being invited to participate in this study that will be carried out at the Barretos Cancer Hospital - Fundação Pio XII. This document is called the “Informed Consent Form” and explains this study and what your participation will be if you accept the invitation. This document also explains the possible risks and benefits if you want to participate, as well as your rights as a research participant. After analyzing the information in this Consent Form and clarifying all your doubts, you will have the necessary knowledge to make a decision about whether or not to participate in this study. Don't be in a rush to decide. If necessary, take it home and read this document with your family or other people you trust.

**WHY IS THIS STUDY BEING DONE?**

When patients need to undergo surgery for their treatment, in most places in the world, the patient is taken to the surgical center on a stretcher, lying down, accompanied by their family member and someone from the nursing team. This study wants to know whether you would feel more comfortable and satisfied if you were taken to have surgery lying down on a stretcher or walking.

**WHAT DOES THIS STUDY WANT TO KNOW?**

This study wants to know whether the patient is less anxious and more comfortable if they go to surgery walking or on a stretcher.

**WHAT WILL HAPPEN TO ME DURING THE STUDY?**

If you agree to participate in the study and sign this document, we will use information about your satisfaction with the way you were taken to the surgery center for the study. In order not to interfere with the answers about your anxiety and preference of mode of transport to the surgical center, the informed consent form is being applied after the journey. A draw was held so that you had an equal chance of falling into both groups. One group was taken to the surgery site by a professional from the nursing team (lying on a stretcher in the company of their family member), and the other group was also accompanied by a professional from the nursing team and their family member, but they came walking. Arriving at the surgical center, the two groups answered two questionnaires, one to find out about their satisfaction with the care offered so far and to find out their satisfaction with the way they were taken to the surgical center. And the other questionnaire is to assess the level of anxiety during the journey to the surgical center. It takes around 10 minutes to answer the two questionnaires, after which you will follow the normal routine of the surgical center, and your participation in this research will end.

**WILL THERE BE ANY RISK OR DISCOMFORT IF I PARTICIPATE IN THE STUDY?**

We cannot rule out the risk of falls during the journey to the surgical center, as some patients will be taken to the surgical center walking, however, to reduce this risk, the patient will be accompanied by their family member and the nursing professional (stretcher) for any support if necessary. To enter the study, all patients will be assessed for fall risk and only those without a fall risk will enter the study. We also cannot rule out the risk of accidental breach of confidentiality, but every care will be taken to ensure this does not happen.

**WILL THERE BE ANY BENEFITS TO ME IF I PARTICIPATE IN THE STUDY?**

It is possible that your participation in this study will not bring any direct benefit to you. But the information obtained at the end of this study could bring benefits to many other patients, if we can identify any difference in the patient's satisfaction and emotional responses (for example, reducing anxiety), whether they are taken to the surgical center walking or on a stretcher, being able to propose a change in the hospital routine.

**WHAT ARE THE OTHER OPTIONS IF I DO NOT PARTICIPATE IN THE STUDY?**

There is no other option to participate in the study. If you do not wish to participate, you have the full right to refuse. Non-participation in the study will not have any consequences or changes to your treatment.

**CAN THE RESEARCH BE SUSPENDED?**

The study can only be suspended after the approval of the Institutional Review Board (IRB) Barretos Cancer Hospital and/or the National Research Ethics Committee (Conep), if applicable, which approved the research, unless closure is for security reasons. In this case, the study may be discontinued without prior analysis by the CEP. However, the researcher must notify the IRB and/or CONEP about the definitive suspension of the study.

**WHAT ARE MY RIGHTS IF I WANT TO PARTICIPATE IN THE STUDY?**

1. You have the right to:
2. Receive study information clearly;
3. Have the opportunity to clarify all your doubts;
4. Have as much time as necessary to decide whether or not you want to participate in the study;
5. Have the freedom to refuse participation in the study, and this will not cause any problems for you;
6. Have the freedom to withdraw and withdraw from the study at any time;
7. Receive assistance with everything necessary if any damage occurs as a result of the study, free of charge, for as long as necessary;
8. Have the right to claim compensation if any damage occurs as a result of the study;
9. Be reimbursed for expenses you and your companion incur as a result of participating in the research, such as transportation and food;
10. Have respected your anonymity (confidentiality);
11. Have your private life respected (privacy);
12. Receive a copy of this document, signed and initialed on all pages by you and the researcher;
13. Have the freedom not to answer questions that bother you;

**IF I HAVE QUESTIONS ABOUT MY RIGHTS OR WANT TO MAKE A COMPLAINT, WHO DO I TALK TO?**

Speak directly to the Barretos Cancer Hospital Institutional Review Board (IRB). Opening hours are from Monday to Thursday, from 8:00 am to 5:00 pm, and Friday, from 8:00 am to 4:00 pm. Lunch hours are from 12:00 to 13:00.

**IF I HAVE QUESTIONS ABOUT THE STUDY, WHO DO I TALK TO?**

Speak directly to the responsible researcher. Contact methods are below:

Barretos Cancer Hospital:

**Researcher name:** Ricardo dos Reis / Gabriela da Silva Oliveira

Contact methods: Gynecology Oncology sector from Monday to Friday from 8:00 am to 5:00 pm, lunch hours are from 12:00 pm to 1:00 pm.

**SUBSCRIPTION FIELD**

|  |  |  |  |  |
| --- | --- | --- | --- | --- |
| Full name of research participant or legal representative |  | Date |  | Signature |

|  |  |  |  |  |
| --- | --- | --- | --- | --- |
| Full name of the researcher |  | Date |  | Signature |

|  |  |  |  |  |
| --- | --- | --- | --- | --- |
| Name in full of the impartial witness (in cases of illiterate, semi-illiterate or visually impaired people) |  | Date |  | Signature |
