# Supplementary material for: Emotional impact according to the way cancer patients are conducted to the surgical center: A randomized clinical trial comparing ambulation to the stretcher
Source: PLoS One. 2025 Apr 22;20(4):e0320856. doi: 10.1371/journal.pone.0320856 (PMC12013869; doi:10.1371/journal.pone.0320856)
Supplement: S2 Protocol — (DOCX) [file pone.0320856.s002.docx]

**HOSPITAL DE CÂNCER DE BARRETOS**

**IMPACTO NOS NÍVEIS DE ANSIEDADE DE ACORDO COM A FORMA DE CONDUÇÃO DE PACIENTES ONCOLÓGICOS ATÉ O CENTRO CIRÚRGICO: ENSAIO CLÍNICO RANDOMIZADO COMPARANDO TRANSPORTE ATRAVÉS DE MACA E DEAMBULAÇÃO.**

**Pesquisador Responsável:** Prof. Dr. Ricardo dos Reis

**Demais Pesquisadores:** Gabriela da Silva Oliveira, Ana Carolina de Matos Magalhães, Marcelo A. Vieira, Carlos Andrade, Audrey Tsunoda, Carlos Eduardo Paiva.

**BARRETOS-SP**

**2020**

**RESUMO**

**Introdução:** O câncer é uma doença extremamente agressiva, e a ansiedade e depressão são consequências que alguns pacientes podem desenvolver, desde o diagnóstico, continuando no decorrer do tratamento. Uma das medidas para o controle do câncer, é a cirurgia para retirada do tumor. O procedimento cirúrgico é frequentemente uma experiência difícil para os pacientes e seus respectivos familiares, e pacientes no período pré-operatório com frequência são encontrados sintomas psicológicos de ansiedade e depressão. Estudos mostram que pacientes acolhidos pelos familiares no período pré-operatório reduzem significativamente os sintomas de ansiedade, medo e pânico frente aqueles que foram unicamente acompanhados pelos profissionais da saúde.**Objetivos:** Analisar a repercussão emocional do paciente diagnosticado com câncer classificado em ECOG PS 0 e 1 de acordo com o tipo de transporte até o centro cirúrgico.**Metodologia:** Ensaio clínico randomizado, com coleta prospectiva, que acontecerá no Hospital de Câncer de Barretos. Para o cálculo amostral, considerando um erro α de 0,05 e um poder 1 – β de 0,80 chegou-se a um tamanho amostral de 176 pacientes, sendo 88 pacientes alocados no grupo maca, e 88 pacientes alocados para o grupo deambulação.**Resultados esperados:** Espera-se encontrar alguma alteração emocional no grau de ansiedade e satisfação dos pacientes conforme modificação do seu tipo de transporte ao centro cirúrgico, sendo possível modificar a rotina hospitalar relacionada a forma de condução destes pacientes.

**Palavras chaves:** Ansiedade, depressão, centro cirúrgico, deambulação, pré-operatório.

1. **INTRODUÇÃO**

Conforme descrito pela Organização Mundial da Saúde (OMS), é indiscutível que o câncer é um problema de saúde pública, principalmente entre os países em desenvolvimento, onde se espera nas próximas décadas que o impacto do câncer na população corresponda a 80% dos mais de 20 milhões de casos novos estimados para 2025([1](#_ENREF_1)).

O câncer é uma doença extremamente agressiva, e a ciência da doença tem grande impacto na vida do paciente, podendo causar alterações físicas e emocionais. A ansiedade e depressão são consequências que alguns pacientes podem desenvolver, desde o diagnóstico, continuando no decorrer do tratamento ([2](#_ENREF_2), [3](#_ENREF_3)).

São vários os tipos de tratamento para o câncer, incluindo além da cirurgia, a quimioterapia, radioterapia, hormonioterapia, imunoterapia e a reabilitação. E alguns tipos de tratamentos trazem inúmeros efeitos colaterais relacionado ao fato de não atingirem exclusivamente as células cancerígenas, dentre eles destacam-se: as náuseas, vômitos, alopecia e queimaduras que enfraquecem o paciente e abalam o seu emocional o que corrobora para a desesperança e o sofrimento do paciente diagnosticado com câncer ([4](#_ENREF_4)).

Uma das medidas para o controle do câncer, é a cirurgia para retirada do tumor, entretanto, embora isso seja frequentemente possível, algumas práticas cirúrgicas podem ter consequências físicas graves, como por exemplo procedimentos cirúrgicos com mutilação, e o medo da intervenção cirúrgica em alguns pacientes com câncer é tão grande, que a ansiedade patológica ou pensamentos distorcidos resultam em recusa do tratamento em mais de 5% dos casos([5](#_ENREF_5), [6](#_ENREF_6)).

Pacientes no período pré-operatório com frequência são encontrados sintomas psicológicos de ansiedade e depressão, e o ideal seria que não houvessem preocupações a não ser aquelas originadas pela própria doença. No entanto, antecipação da dor, separação da família, perda da independência, medo da incapacitação além do medo do procedimento em si e da morte, acabam sendo fatores que com frequência desencadeiam estes sintomas durante este período ([7](#_ENREF_7)).

Em um estudo prévio observou-se que a ansiedade no pré-operatório, estava presente em cerca de 80% dos pacientes adultos que aguardavam algum tipo de cirurgia. Frente a isso, a ansiedade merece a devida atenção da equipe de saúde, pois pode influenciar na resposta do paciente ao tratamento e causar efeitos negativos em sua recuperação pós-operatória,além do mais, a ansiedade acarreta alterações fisiológicas, como taquicardia e hipertensão arterial, com consequente aumento do consumo de oxigênio e piora da evolução da doença ([8](#_ENREF_8)).

O procedimento cirúrgico é frequentemente uma experiência difícil para os pacientes e seus respectivos familiares, e requer uma série de mobilizações afetivas para lidar com a ansiedade e o estresse da situação. Qualquer ato cirúrgico é considerado uma situação crítica capaz de despertar comportamentos individuais, influenciada por múltiplos fatores físicos e emocionais ([9](#_ENREF_9)).

Uma das estratégias utilizadas para diminuir os níveis de ansiedade e depressão durante o período pré-operatório é o fornecimento de informações sobre as condições de saúde, procedimentos realizados e a participação do familiar neste momento ([9](#_ENREF_9)).

Estudos mostram que pacientes acolhidos pelos familiares no período pré-operatório reduzem significativamente os sintomas de ansiedade, medo e pânico frente aqueles que foram unicamente acompanhados pelos profissionais da saúde. A presença do familiar reduz principalmente o sentimento de ansiedade do pré-operatório, caracterizado como um sentimento de medo e apreensão do desconhecido, ao contrário do que acontece quando há a presença única do profissional de enfermagem, aumentando os índices de ansiedade em relação aos pacientes que não foram acompanhados por seus familiares. Dessa forma, é possível ver a relação imprescindível entre a presença familiar e a redução da ansiedade num período tão crítico que é o pré-operatório([8](#_ENREF_8)).

Outro ponto importante é que na literatura é muito comum serem encontrados artigos que definem a importância de um transporte intra-hospitalar para fins diagnósticos ou terapêuticos de pacientes críticos, dentre eles, aqueles que apresentam algum tipo de câncer em tratamento na Unidade de Terapia Intensiva (UTI). O translado destes pacientes depende diretamente do planejamento e da atuação organizada da equipe multidisciplinar, assim como a escolha de equipamentos indispensáveis, sendo necessário porque pacientes em estado crítico são mais suscetíveis a alterações fisiológicas podendo causar complicações no decorrer do caminho. Porém, pouco é estudado sobre o transporte para o centro cirúrgico quando o paciente se encontra em estado não crítico([8](#_ENREF_8), [10](#_ENREF_10), [11](#_ENREF_11)).

Kojima e*t al.* ([12](#_ENREF_12))realizou um estudo entre outubro de 1998 a setembro de 2000 com pacientes de 15 a 80 anos que seriam submetidos a algum tipo de cirurgia, porém no estudo não foram especificados as doenças prévias e nem o motivo da da cirurgia, dando devida importância apenas a forma de condução ao centro cirúrgico: uma através do uso de maca e a outra através da deambulação, analisando também o grau de consciência dos pacientes perante os medicamentos usados, chegando ao seguinte resultado: pacientes que vão caminhando sentem-se mais tranquilos no pré-operatório. O uso da maca os deixam mais ansiosos, angustiados e até mesmo inferiorizados ao conversar com outras pessoas já que permanecem deitados e chamam mais atenção por onde passam. Tudo isto, reflete o estresse causado por esse tipo de transporte, entendendo-se que a deambulação reduz o nível de ansiedade causada por esse tipo de transporte.Entretanto, neste estudo, não são especificadas as doenças, como o câncer, o grau de limitação dos pacientes e nem a classificação em score PS dos pacientes submetidos ao ato cirúrgico. Outro ponto importante, é o amplo espectro de faixa etária dos pacientes analisados pelo estudo, uma vez que engloba desde adolescentes até idosos, gerando muitas variáveis para uma análise veraz dos resultados.

1. **JUSTIFICATIVA**

Pacientes com câncer apresentam alterações emocionais a partir do momento de seu diagnóstico e no decorrer de todo o tratamento, por esta razão, o estudo deseja observar se há alguma alteração das respostas emocionais, do grau de ansiedade e da satisfação dos pacientes adultos com câncer classificados em PS 0 e 1 conforme a mudança do tipo de transporte ao centro cirúrgico, substituindo o uso da maca (modelo tradicional) pela deambulação. Além disso, há um déficit de trabalhos publicados na literatura sobre este tema, principalmente em pacientes com câncer, e conforme os achados finais, poder mudar a prática diária em relação a forma de condução destes pacientes até o centro cirúrgico, diminuindo os níveis de ansiedade e com isso repercutindo numa melhor recuperação do paciente.

- 1. **Fator em Estudo**

Forma de condução ao centro cirúrgico (maca versus deambulação)

- 1. **Desfecho primário**

Impacto emocional conforme forma de condução ao centro cirúrgico.

1. **OBJETIVOS**
   1. **Objetivo primário**

**3.1.** Determinar os níveis de ansiedade do paciente diagnosticado com câncer de acordo com o tipo de transporte até o centro cirúrgico.

**Objetivos secundários**

- - 1. Analisar se o tipo de transporte do paciente classificado em PS 0 e 1 tem influência no grau de depressão.

**3.2.2** Analisar se o tipo de transporte do paciente classificado em PS 0 e 1 tem influência no grau de satisfação do paciente.

**3.2.3** Analisar a ansiedade do paciente de acordo com o tipo de transporte, conforme o gênero.

**3.2.4** Analisar a ansiedade do paciente de acordo com o tipo de transporte, conforme a nível sócio econômico.

1. **METODOLOGIA**

**4.1 Delineamento**

Ensaio clínico randomizado, com coleta prospectiva, para analisar a repercussão emocional do paciente diagnosticado com câncer classificado em ECOG PS 0 e 1 de acordo com o tipo de transporte até o centro cirúrgico.

**4.2 Amostra**

Para seleção destes pacientes, utilizaremos a escala de avaliação do risco de quedas adotada na rotina hospitalar para pacientes acima de 60 anos de idade, juntamente com a escala de *Eastern Cooperative Oncology Group (ECOG PS)* (Tabela 1)*,* sendo classificado apenas pacientes sem risco ou com risco mínimo, segundo a escala de risco de quedas (Tabela 2), e pacientes com PS 0 e 1.

**Tabela 1 -ECOG Performance Status** (Grupo de Oncologia *Eastern Cooperative, Robert L. Comis, MD.)*

| Grau | **ECOG PERFORMANCE STATUS** |
| --- | --- |
| 0 | Totalmente ativo, capaz de desenvolver todo seu desempenho, sem qualquer restrição. |
| 1 | Restrições nas atividades extenuantes, porém mantendo a normalidade em sua prática diária. |
| 2 | Ainda conserva sua capacidade de autocuidado, mas é incapaz de realizar qualquer atividade de trabalho, e se realiza, é em menos de 50% das horas de vigília. |
| 3 | Capacidade limitada de autocuidado; confinado a uma cama ou cadeira em 50% do tempo de vigília. |
| 4 | Completamente inabilitado, sem poder exercer o autocuidado, totalmente confinado a uma cama ou cadeira. |
| 5 | Óbito |

**Tabela 2: -** ESCALA PARA AVALIAÇÃO DO RISCO DE QUEDA (Adaptada Jonhs Hopkins 2007)

| Zero = Sem risco 1-5 = Baixo Risco 6-13 = Moderado Risco > 13 = Alto Risco | |
| --- | --- |
| **Fator de Risco** | **Pontuação** |
| 1. **Idade** (selecionar 1 opção) |  |
| - 60 – 69 anos | 1 |
| - 70 – 79 anos | 2 |
| - Maior ou igual 80 anos | 3 |
| 1. **História de queda** |  |
| - Queda nos últimos 6 meses antes da internação | 5 |
| 1. **Eliminações** (selecionar 1 opção): |  |
| - Incontinência | 2 |
| - Urgência ou alteração da frequência | 2 |
| - Urgência/ alteração da frequência e incontinência | 4 |
| 1. **Medicações** |  |
| - Uso de 1 das opções | 3 |
| - Uso de 2 ou mais opções | 5 |
| - Submetido a procedimentos com sedativos nas últimas 24 horas | 7 |
| 1. **Uso de Equipamentos:** qualquer equipamento usado pelo paciente, tais como cateteres, punção venosa, entre outros (selecionar 1 opção) |  |
| - Presença de 1 equipamento | 1 |
| - Presença de 2 equipamentos | 2 |
| - Presença de 3 ou mais equipamentos | 3 |
| 1. **Mobilidade** (permitido seleção múltipla) |  |
| - Necessita de auxilio ou supervisão para movimentação, transferência ou deambulação | 2 |
| - Marcha instável | 2 |
| - Deficiência visual e/ou auditiva que afeta a movimentação | 2 |
| 1. **Cognitivo** (permitido seleção múltipla): |  |
| - Alteração da consciência relacionada ao ambiente | 1 |
| - Impulsivo | 2 |
| - Falta de compreensão de suas limitações físicas e cognitivas | 4 |
| 1. **Condições especiais:** De acordo com a lista: | Não / Sim |

Condições de saúde e presença de doenças crônicas – devem estar ligados ao item 8 da escala:

Acidente vascular cerebral prévio, hipotensão postural, tonteira, baixo índice de massa corpórea, anemias, insônias, incontinência ou urgência miccional, artrite, osteoporose, alterações metabólicas (ex: hipoglicemia).

**Tabela 3 -** Medicamentos relacionados ao uso de média item 4.

| Atenolol | Dimenidrinato | Insulina Humana Regular | Nalbufina | Sultamicilina |
| --- | --- | --- | --- | --- |
| Atracúrio | Dipiridamol | Insulina NPH | Naloxona | Suxametônio |
| Atropina | Dipirona | Iodeto de Potássio | Naproxeno | Tracolimo |
| Azul de metinelo | Dobutamina | Iodixanol (bula visipaque) | Neostigmina | Tacrolimo Tópico |
| Benzidamina | Dopamina | Ioversol | Nifedipino | Teicoplanina |
| Betametasona | Droperidol | Ipratrópio | Nimesulida | Terbutalina |
| Bisacodil | Epinefrina | Ipratrópio + Fenoterol | Nitroglicerina | Tiabendazol |
| Bromoprida | Ertapenem |  | Nitroprussiato | Tiopental |
| Budesonida | Escopolamina | Isossorbida | Norfloxacino | Tramadol |
| Bupivacaina | Espironolactona | Itraconazol | Nortripitilina | Valproato de Sódio |
| Captopril | Estreptoquinase | Ivermectina | Octreotida | Vancomicina |
| Carbamazepina | Etilefrina | Lamivudina | Omeprazol | Varfarina |
| Carvedilol | Etomidato | Levofloxacino | Ondansetrona | Vasopressina |
| Ceftriaxona | Fenazopiridina | Levomepromazina | Pancurônio | Verapamil |
| Cetamina | Fenitoína | Levotiroxina | Paracetamol | Voriconazol |
| Cetoprofeno | Fenobarbital | Lindocaína | Paroxetina |  |
| Cetorolaco de trometamina | Fenoterol | Lindocaína + Epinefrina | Petidina |  |
| Ciclosporina | Fentalina | Lindocaína 25 mg/g + Prilocaína 25 mg/g 5g | Piperacilina |  |
| Cirpofloxacino | Fitomenadiona (vitamina K) | Loperamida | Polimexina B |  |

**4.3 Cálculo amostral**

Com base nos resultados encontrados por Kojima e*t al.* ([12](#_ENREF_12)) foi realizado o cálculo amostral para comparação de proporções. Considerando um erro α de 0,05 e um poder 1 – β de 0,80 chegou-se a um tamanho amostral de 176 pacientes, sendo 88 pacientes alocados no grupo maca, e 88 pacientes alocados para o grupo deambulação.

**4.4 Critérios de Elegibilidade**

- Pacientes com diagnóstico de câncer
- Idade igual ou superior a 18 anos, indo até 70 anos
- Ambos os sexos
- Classificados em score PS 0 ou 1
- Pacientes que sejam classificados em Risco Zero ou Baixo Risco pela escala de avaliação de risco de quedas
- Indicação de cirurgia eletiva

**4.5 Critérios de não elegibilidade**

- Pacientes que momentos antes da cirurgia apresentarem algum evento adverso que possa pôr em risco a deambulação até o centro cirúrgico.
- Pacientes que tenham deficiência física e necessitam de auxílio para deambular.
- Pacientes que tem indicação de leito na Unidade de Terapia Intensiva (UTI)
- Pacientes que tenham transtornos psiquiátricos previamente diagnosticados (depressão grave, transtorno de ansiedade, pânico, dentre outros).
- Pacientes ECOG > 1.
- Pacientes que não apresentarem um acompanhante no dia da cirurgia.
- Pacientes em uso de medicações ansiolíticas e antidepressivas.

**4.6 Coleta de Dados**

Será confeccionado um instrumento de pesquisa na plataforma de dados REDCap. Nela, coletaremos dados sócio demográficos como: data de admissão, data de nascimento, peso, altura, sexo, grupo étnico, residência atual, telefone para contato, nome do acompanhante e seu grau de parentesco, nível sócio econômico, escolaridade. Utilizaremos o documento fonte (prontuário) para informações como: classificação dos tumores malignos (TNM), classificação da performance status de acordo com as comorbidades, classificação da escala de risco de queda, que é realizada no momento da internação do paciente pelo funcionário responsável pelo mesmo, medicamentos em uso, outras cirurgias, exames complementares confirmando a presença do câncer, o tipo e o local da neoplasia, data da cirurgia, porte cirúrgico.

Os pacientes serão identificados como possíveis participantes para o estudo no momento de sua internação. Preenchendo todos os critérios de elegibilidade o mesmo será randomizado de forma aleatória através da plataforma REDCap, e será realizado a orientação de qual grupo o paciente caiu, para o profissional de enfermagem (maqueiro) que o conduzirá até o centro cirúrgico.

O grupo 1 será acolhido em seu quarto por um técnico de enfermagem, um familiar e pelo maqueiro que o levará deitado em uma maca até o centro cirúrgico, acompanhado de seu familiar, onde será recebido por um profissional de enfermagem que fará os procedimentos padrões de centro cirúrgico. Já o grupo 2 sairá de seu quarto e irá ao centro cirúrgico deambulando junto a um familiar e o maqueiro que o acompanhará durante o percurso. Os pacientes randomizados para o grupo 1 deverão ser conduzidos ao centro cirúrgico com a roupa hospitalar, já os pacientes randomizados para o grupo 2, deverão ser conduzidos ao centro cirúrgico com a roupa própria. Ao adentrar o centro cirúrgico, ambos os pacientes do grupo 1 e 2 responderão ao questionário escala HAD – avaliação do nível de ansiedade e depressão, de fácil manuseio e rápida execução que foi desenvolvida para detectar estados de ansiedade e depressão em pacientes fisicamente doentes, que podem responde-la sozinhos ([13](#_ENREF_13), [14](#_ENREF_14)) (ANEXO 1), e ao Questionário de Avaliação da Satisfação com os Cuidados em Saúde em Cirurgia (Sati-Cir) (APENDICE1), questionário este desenvolvido para o presente estudo a fim de avaliar a satisfação do paciente com relação aos cuidados recebidos no hospital, e avaliar a satisfação com a forma de condução do mesmo até o centro cirúrgico. Para este questionário, será realizado um teste piloto com 20 pacientes que terão sua forma de condução ao centro cirúrgico randomizada de forma aleatória via RedCap, a fim de analisar o entendimento dos pacientes com relação aos questionamentos feitos.

Após a aplicação de ambos os questionários, será realizada a aplicação do Termo de Consentimento Livre e Esclarecido (TCLE) do referente estudo. A aplicação do TCLE será realizada após o transporte e aplicação dos questionários no centro cirúrgico, para que não ocorra um viés do participante na forma em que o mesmo responderá os questionários, para não o induzir a acreditar que ser levado ao centro cirúrgico deambulando seja mais confortável e provoque mais satisfação.

Ambos os questionários serão utilizados para avaliar os achados quanto as alterações emocionais, grau de ansiedade e satisfação vividas pelo paciente naquele momento, os questionários são autoaplicáveis ou poderão ser aplicados ao paciente por membros do Núcleo de Apoio ao Pesquisador, e aluna de iniciação científica do presente estudo.

**4.7 Local do estudo**

O presente projeto abrangerá um estudo de coleta prospectiva dos pacientes com indicação cirúrgica de forma eletiva no Hospital de Câncer de Barretos, centro de referência nacional no tratamento de pacientes oncológicos.

**4.8 Randomização**

A randomização será gerada através da plataforma – REDCap - criando uma lista aleatória de entrada no estudo, separando em grupo 1 - controle (pacientes que serão transportados ao centro cirúrgico pelo método tradicional, de maca) e grupo 2 - experimental (pacientes que irão deambulando até o centro cirúrgico), ambos os grupos acompanhados por seus familiares. Desta forma, todos os pacientes terão igual chance de serem incluídos nos dois grupos.

**4.9 Análise estatística**

Inicialmente serão descritos os dados de nossa amostra através de medidas de tendência central (média e mediana) e dispersão (desvio padrão e quartis) para todas as variáveis do tipo quantitativo. Já para as variáveis do tipo qualitativo, iremos utilizar tabelas contendo os valores absolutos e relativos de cada categoria das variáveis. Posteriormente, com a finalidade de observar a diferença entre os grupos de estudo (Maca x Deambulando) em relação a repercussão emocional, grau de satisfação e ansiedade, utilizaremos testes estatísticos considerando a significância de 0,05. Caso as variáveis a serem relacionadas apresentem distribuição Normal, faremos uso do Teste T, e caso contrário, aplicaremos o Teste de Mann-Whitney. Já para as variáveis categóricas, empregaremos os testes Qui-Quadrado ou Exato de Fischer, dependendo das características dos dados. Para tais análises, utilizaremos o software SPSS versão 21, e o auxílio do Núcleo de Epidemiologia e Bioestatística (NEB) do Hospital de Câncer de Barretos.

**5. QUESTÕES ÉTICAS**

Este projeto está de acordo com a ética e metodologicamente com as Diretrizes e Normas Regulamentadoras de Pesquisa envolvendo Seres Humanos (Resolução 466/12 do Conselho Nacional de Saúde) e será submetido ao Comitê de Ética e Pesquisa (CEP) do Hospital de Câncer de Barretos. Os pacientes assinarão um termo de consentimento livre e esclarecido para participação no estudo (ANEXO 3). A não assinatura do termo implicará na não participação do paciente no estudo, porém não interferirá no tratamento do mesmo na instituição.

**5.1 Riscos ao Participante**

Há o risco de quebra da confidencialidade de dados entre os membros do estudo, mas este será evitado ao máximo, assim como o risco mínimo de quedas, já que um grupo em específico será conduzido ao centro cirúrgico deambulando. E para minimizar estes riscos, realizaremos a escala de risco de quedas utilizada na rotina do hospital, não expondo nenhum paciente à intercorrências. O paciente será acompanhado pelo maqueiro e seu familiar até o centro cirúrgico, para possível apoio caso o mesmo sinta-se indisposto durante este percurso.

**5.2 Benefício ao participante**

Consideramos encontrar uma relação entre a forma de condução ao centro cirúrgico destes pacientes devidamente classificados em ECOG PS 0 e 1, com o grau de ansiedade apresentado pelo mesmo antes do ato cirúrgico. Podendo alterar a rotina de transporte dos pacientes até o centro cirúrgico, visto que são pacientes em bom estado físico, podendo acrescer algum benefício psicológico, afastando a ideia de enfermidade e incapacitação perante este simples percurso.

**6. ORÇAMENTO**

Os gastos deste estudo serão relacionados a impressão de termos de consentimentos livres e esclarecidos, banco de dados e do questionário, portanto, serão necessários apenas folhas brancas de sulfite A4 e a reposição do cartucho preto da impressora, que serão financiados com recursos particulares do pesquisador, que ficarão em torno de R$200,00.

**7. CRONOGRAMA**

| **ATIVIDADES** | **2018** | **2019** | | **2020** | | | **2021** | |
| --- | --- | --- | --- | --- | --- | --- | --- | --- |
| **PERÍODO** | **P1** | **P1** | **P2** | **P1** | **P2** | **P3** | **P1** | **P2** |
| Comissão de Ética | **X** |  |  |  |  |  |  |  |
| Aprovação *Clinical Trials* |  | **X** |  |  |  |  |  |  |
| Coleta de Dados |  | **X** | **X** | **X** | **X** | **X** |  |  |
| Disciplinas |  |  | **X** | **X** | **X** | **X** | **X** |  |
| Bancas de acompanhamento |  |  |  | **X** |  | **X** |  | **X** |
| Análise Estatística |  |  |  |  |  |  | **X** | **X** |
| Discussão dos Resultados |  |  |  |  |  |  | **X** | **X** |
| Redação da tese |  |  |  |  |  |  |  | **X** |
| Defesa |  |  |  |  |  |  |  | **X** |

*P: período de 4 meses*

**REFERÊNCIAS BIBLIOGRÁFICAS**

1. Estimativa 2016 - Incidência de Câncer no Brasil Rio de Janeiro2015 [Available from: <http://www.inca.gov.br>.

2. Bailey RK, Geyen DJ, Scott-Gurnell K, Hipolito MM, Bailey TA, Beal JM. Understanding and treating depression among cancer patients. Int J Gynecol Cancer. 2005;15(2):203-8.

3. Jenkins PL, May VE, Hughes LE. Psychological morbidity associated with local recurrence of breast cancer. Int J Psychiatry Med. 1991;21(2):149-55.

4. Barbosa LNF SD, Amaral MX, Gonçalves AJ, Bruscato WL. Repercussões psicossociais em pacientes submetidos a laringectomia total por câncer de laringe: Um estudo clínico-qualitativo. Revista da Sociedade Brasileira de Psicologia Hospitalar. 2004(7(1)):45-58.

5. Bottomley A. Psychosocial problems in cancer care: a brief review of common problems. J Psychiatr Ment Health Nurs. 1997;4(5):323-31.

6. Braz DS, Ribas MM, Dedivitis RA, Nishimoto IN, Barros AP. Quality of life and depression in patients undergoing total and partial laryngectomy. Clinics (Sao Paulo). 2005;60(2):135-42.

7. Maranets I, Kain ZN. Preoperative anxiety and intraoperative anesthetic requirements. Anesth Analg. 1999;89(6):1346-51.

8. Assis CC, Lopes Jde L, Nogueira-Martins LA, de Barros AL. [Embracement and anxiety symptoms in patients before cardiac surgery]. Rev Bras Enferm. 2014;67(3):401-7.

9. Santos MAd, Rossi LA, Paiva L, Dantas RAS, Pompeo DA, Machado ECB. Medida da ansiedade e depressão em pacientes no pré-operatório de cirurgias eletivas. Rev eletrônica enferm. 2012;14(4):922-7.

10. Kelly CM, Shahrokni A. Moving beyond Karnofsky and ECOG Performance Status Assessments with New Technologies. J Oncol. 2016;2016:6186543.

11. Rossi L SM. Repercussões psicológicas do adoecimento e tratamento em mulheres acometidas pelo câncer de mama. Psicologia, Ciência e Profissão. 2003(23(4)):32-41.

12. Kojima Y, Ina H, Fujita T, Mitono H. Relieving anxiety by entering the operating room on foot. Can J Anaesth. 2002;49(8):885-6.

13. Magalhaes Filho LL, Segurado A, Marcolino JA, Mathias LA. [Impact of preanesthetic evaluation on anxiety and depression in cancer patients undergoing surgery.]. Rev Bras Anestesiol. 2006;56(2):126-36.

14. Marcolino JA, Mathias LA, Piccinini Filho L, Guaratini AA, Suzuki FM, Alli LA. Hospital Anxiety and Depression Scale: a study on the validation of the criteria and reliability on preoperative patients. Rev Bras Anestesiol. 2007;57(1):52-62.

**ANEXO 1. Escala HAD – Avaliação do Nível de Ansiedade e Depressão (**[**14**](#_ENREF_14)**).**

Este questionário ajudará o seu médico a saber como você está se sentindo. Leia todas as frases. Marque com um “X” a resposta que melhor corresponder a como você tem se sentido na ÚLTIMA SEMANA. Não é preciso ficar pensando muito em cada questão. Neste questionário as respostas espontâneas têm mais valor do que aquelas em que se pensa muito. Marque apenas uma resposta para cada pergunta.

**A** 1) Eu me sinto tenso ou contraído:

3 ( ) A maior parte do tempo

2 ( ) Boa parte do tempo

1 ( ) De vez em quando

0 ( ) Nunca

**D** 2) Eu ainda sinto gosto pelas mesmas coisas de antes:

0 ( ) Sim, do mesmo jeito que antes

1 ( ) Não tanto quanto antes

2 ( ) Só um pouco

3 ( ) Já não sinto mais prazer em nada

**A** 3) Eu sinto uma espécie de medo, como se alguma coisa ruim fosse acontecer:

3 ( ) Sim, e de um jeito muito forte

2 ( ) Sim, mas não tão forte

1 ( ) Um pouco, mas isso não me preocupa

0 ( ) Não sinto nada disso

**D** 4) Dou risada e me divirto quando vejo coisas engraçadas:

0 ( ) Do mesmo jeito que antes

1 ( ) Atualmente um pouco menos

2 ( ) Atualmente bem menos

3 ( ) Não consigo mais

**A**5) Estou com a cabeça cheia de preocupações:

3 ( ) A maior parte do tempo

2 ( ) Boa parte do tempo

1 ( ) De vez em quando

0 ( ) Raramente

**D**6) Eu me sinto alegre:

3 ( ) Nunca

2 ( ) Poucas vezes

1 ( ) Muitas vezes

0 ( ) A maior parte do tempo

**A** 7) Consigo ficar sentado à vontade e me sentir relaxado:

0 ( ) Sim, quase sempre

1 ( ) Muitas vezes

2 ( ) Poucas vezes

3 ( ) Nunca

**D** 8) Eu estou lento para pensar e fazer as coisas:

3 ( ) Quase sempre

2 ( ) Muitas vezes

1 ( ) De vez em quando

0 ( ) Nunca

**A** 9) Eu tenho uma sensação ruim de medo, como um frio na barriga ou um aperto no estômago:

0 ( ) Nunca

1 ( ) De vez em quando

2 ( ) Muitas vezes

3 ( ) Quase sempre

**D** 10) Eu perdi o interesse em cuidar da minha aparência:

3 ( ) Completamente

2( ) Não estou mais me cuidando como deveria

1 ( ) Talvez não tanto quanto antes

0 ( ) Me cuido do mesmo jeito que antes

**A** 11) Eu me sinto inquieto, como se eu não pudesse ficar parado em lugar nenhum:

3 ( ) Sim, demais

2 ( ) Bastante

1 ( ) Um pouco

0 ( ) Não me sinto assim

**D** 12) Fico esperando animado as coisas boas que estão por vir:

0 ( ) Do mesmo jeito que antes

1 ( ) Um pouco menos do que antes

2 ( ) Bem menos do que antes

3 ( ) Quase nunca

**A** 13) De repente, tenho a sensação de entrar em pânico:

3 ( ) A quase todo momento

2 ( ) Várias vezes

1 ( ) De vez em quando

0 ( ) Não sinto isso

**D** 14) Consigo sentir prazer quando assisto a um bom programa de televisão, de rádio ou quando leio alguma coisa:

0 ( ) Quase sempre

1 ( ) Várias vezes

2 ( ) Poucas vezes

3 ( ) Quase nunca

HAD-ansiedade: sem ansiedade de 0 a 8, com ansiedade ≥ 9.

HAD-depressão: sem depressão de 0 a 8, com depressão ≥ 9.

**APENDICE1: Questionário de Avaliação da Satisfação com os Cuidados em Saúde em Cirurgia (Sati-Cir).**

Este questionário tem o objetivo de avaliar sua SATISFAÇÃO com os cuidados de saúde recebidos neste hospital, principalmente em relação às questões relativas à cirurgia. Ele é composto de duas partes: a Parte A contem 8 itens com 5 opções de resposta, variando de 1 (muito satisfeito) a 5 (muito insatisfeito). Na parte B, duas questões complementares avaliam sua satisfação em relação aos cuidados recebidos, com opções de respostas qualitativas (com explicações mais detalhadas).

**Parte A**

|  | Muito Satisfeito | Satisfeito | Indiferente | Insatisfeito | Muito Insatisfeito |  |  |  |  |  |
| --- | --- | --- | --- | --- | --- | --- | --- | --- | --- | --- |
| 1. *Qual seu grau de satisfação em relação ao esclarecimento de dúvidas sobre sua doença?* | 1 | 2 | 3 | 4 | 5 |  |  |  |  |  |
| 1. *Qual seu grau de satisfação em relação a sua participação nas decisões relacionadas ao seu tratamento?* | 1 | 2 | 3 | 4 | 5 |  |  |  |  |  |
| 1. *Qual seu grau de satisfação em relação as oportunidades que a equipe lhe dá para fazer perguntas?* | 1 | 2 | 3 | 4 | 5 |  |  |  |  |  |
| 1. *Qual seu grau de satisfação em relação a forma que consegue uma informação quando pede uma?* | 1 | 2 | 3 | 4 | 5 |  |  |  |  |  |
| 1. *Qual seu grau de satisfação em relação aos cuidados da equipe de enfermagem para preservar sua privacidade?* | 1 | 2 | 3 | 4 | 5 |  |  |  |  |  |
| 1. *Qual seu grau de satisfação em relação ao tempo que esperou até ser internado?* | 1 | 2 | 3 | 4 | 5 |  |  |  |  |  |
| 1. *Qual seu grau de satisfação em relação ao tempo que esperou até o momento da cirurgia?* 2. *Como você foi conduzido ao centro cirúrgico:* | 1 | 2  Maca | 3 | 4  Deambulando | 5 |  |  |  |  |  |
| 1. *Qual seu grau de satisfação em relação a forma como foi conduzido até o centro cirúrgico?* 2. *Como você foi conduzido ao centro cirúrgico:* | 1 | 2  Pijama | 3 | 4  Roupa própria | 5 | 1 | 2 | 3 | 4 | 5 |
| 1. *Qual o seu grau de satisfação em relação a vestimenta que você foi conduzido ao centro cirúrgico?* | 1 | 2 | 3 | 4 | 5 |  |  |  |  |  |

**Parte B**

**Em relação à vestimenta que você foi conduzido (a) ao centro cirúrgico, responda:**

Você achou que sua **dignidade/privacidade** foi preservada em relação a vestimenta que você foi conduzido até o centro cirúrgico?

( ) Sim

( ) Não Porque:________________________________________________________________

**Em relação à forma com que foi conduzido (a) ao centro cirúrgico, responda:**

Você achou que sua **autonomia** foi preservada em relação ao modo como você foi conduzido até o centro cirúrgico?

( ) Sim

( ) Não Porque:________________________________________________________________

Se você pudesse escolher a forma de ser conduzido até o centro cirúrgico, qual seria?

( ) Maca

( ) Cadeira de Rodas

( ) Caminhando

**ANEXO 2 (Termo de Consentimento Livre e Esclarecido)**

**TERMO DE CONSENTIMENTO LIVRE E ESCLARECIDO (TCLE)**

**PARA PARTICIPAÇÃO EM PESQUISA**

**TÍTULO DO ESTUDO**: **IMPACTO NOS NÍVEIS DE ANSIEDADE DE ACORDO COM A FORMA DE CONDUÇÃO DE PACIENTES ONCOLÓGICOS ATÉ O CENTRO CIRÚRGICO: ENSAIO CLÍNICO RANDOMIZADO COMPARANDO TRANSPORTE ATRAVÉS DE MACA E DEAMBULAÇÃO.**

**PESQUISADORES:**

Ricardo dos Reis, Gabriela da Silva Oliveira, Ana Carolina de Matos Magalhães, Marcelo A. Vieira, Carlos Andrade, Audrey Tsunoda, Carlos Eduardo Paiva.

**O QUE É ESTE DOCUMENTO?**

Você está sendo convidado (a) a participar deste estudo que será realizado no Hospital de Câncer de Barretos - Fundação Pio XII. Este documento é chamado de “Termo de Consentimento Livre e Esclarecido” e explica este estudo e qual será a sua participação, caso você aceite o convite. Este documento também fala os possíveis riscos e benefícios se você quiser participar, além de dizer os seus direitos como participante de pesquisa. Após analisar as informações deste Termo de Consentimento e esclarecer todas as suas dúvidas, você terá o conhecimento necessário para tomar uma decisão sobre sua participação ou não neste estudo. Não tenha pressa para decidir. Se for preciso, leve para a casa e leia este documento com os seus familiares ou outras pessoas que são de sua confiança.

**POR QUE ESTE ESTUDO ESTÁ SENDO FEITO?**

Quando os pacientes precisam fazer uma cirurgia para seu tratamento, na maioria dos locais do mundo, o paciente é levado para o centro cirúrgico de maca, deitado, acompanhado de seu familiar e alguém da enfermagem. Este estudo quer saber se você se sentiria mais confortável e satisfeito se fosse levado para fazer a cirurgia deitado em uma maca ou caminhando.

**O QUE ESTE ESTUDO QUER SABER?**

Este estudo quer saber se o paciente fica menos ansioso e mais confortável se for para a cirurgia andando, ou de maca.

**O QUE ACONTECERÁ COMIGO DURANTE O ESTUDO?**

Se você aceitar participar do estudo e assinar este documento, utilizaremos as informações sobre sua satisfação com o modo que foi levado ao centro cirúrgico para o estudo. Para não interferir nas respostas sobre sua ansiedade e preferência do modo de transporte ao centro cirúrgico, a aplicação do TCLE está sendo feita após o trajeto. Foi realizado um sorteio para que você tivesse chances iguais de cair nos dois grupos. Um grupo foi levado para o local da cirurgia por um profissional da equipe de enfermagem (deitado em uma maca na companhia do familiar), e o outro grupo foi também acompanhado por um profissional da equipe de enfermagem e seu familiar, porém veio caminhando. Chegando ao centro cirúrgico, os dois grupos responderam a dois questionários, um para sabermos sobre a satisfação com relação aos cuidados que oferecidos até o momento e para saber a satisfação com a forma que foi levado ao centro cirúrgico. E o outro questionário é para avaliar o nível de ansiedade durante o trajeto até o centro cirúrgico. Para responder os dois questionários leva cerca de 10 minutos, e após você seguira a rotina normal do centro cirúrgico, e se encerra sua participação nesta pesquisa.

**HAVERÁ ALGUM RISCO OU DESCONFORTO SE EU PARTICIPAR DO ESTUDO?**

Não podemos descartar o risco de quedas durante o caminho até o centro cirúrgico, visto que alguns pacientes serão levados ao centro cirúrgico caminhando, porém, para diminuir esse risco o paciente será acompanhado pelo seu familiar e pelo profissional da enfermagem (maqueiro), para qualquer suporte se for necessário. Para entrar no estudo, todos os pacientes serão avaliados para o risco de queda e só aqueles sem risco de queda entrarão no estudo. Também não podemos descartar o risco de quebra acidental de sigilo, porém todo cuidado será tomado para que isto não aconteça.

**HAVERÁ ALGUM BENEFÍCIO PARA MIM SE EU PARTICIPAR DO ESTUDO?**

É possível que sua participação neste estudo não traga nenhum benefício direto para você. Mas as informações obtidas ao final deste estudo poderão trazer benefícios a muitos outros pacientes, se conseguirmos identificar alguma diferença na satisfação e nas respostas emocionais do paciente (por exemplo diminuir a ansiedade), no fato de ser levado ao centro cirúrgico caminhando ou de maca, podendo propor uma mudança na rotina do hospital.

**QUAIS SÃO AS OUTRAS OPÇÕES SE EU NÃO PARTICIPAR DO ESTUDO?**

Não há outra opção de participação do estudo. Caso não queira participar tem o total direito de recusa. A não participação no estudo não trará nenhuma consequência ou mudança no seu tratamento.

**A PESQUISA PODE SER SUSPENSA?**

O estudo somente poderá ser suspenso após a anuência do Comitê de Ética em Pesquisa do Hospital de Câncer de Barretos (CEP) e/ou da Comissão Nacional de Ética em Pesquisa (Conep), se for o caso, que aprovou a realização da pesquisa, a menos que o encerramento se dê por razões de segurança. Nesse caso, o estudo poderá ser descontinuado sem prévia análise do CEP. Contudo, o pesquisador deve notificar o CEP e/ou a CONEP sobre a suspensão definitiva do estudo.

**QUAIS SÃO OS MEUS DIREITOS SE EU QUISER PARTICIPAR DO ESTUDO?**

Você tem direito a:

1. Receber as informações do estudo de forma clara;
2. Ter oportunidade de esclarecer todas as suas dúvidas;
3. Ter o tempo que for necessário para decidir se quer ou não participar do estudo;
4. Ter liberdade para recusar a participação no estudo, e isto não trará qualquer de problema para você;
5. Ter liberdade para desistir e se retirar do estudo a qualquer momento;
6. Ter assistência a tudo o que for necessário se ocorrer algum dano decorrente do estudo, de forma gratuita, pelo tempo que for preciso;
7. Ter direito a reclamar indenização se ocorrer algum dano decorrente do estudo;
8. Ser ressarcido pelos gastos que você e seu acompanhante tiverem por causa da participação na pesquisa, como por exemplo, transporte e alimentação;
9. Ter respeitado o seu anonimato (confidencialidade);
10. Ter respeitada a sua vida privada (privacidade);
11. Receber uma via deste documento, assinada e rubricada em todas as páginas por você e pelo pesquisador;
12. Ter liberdade para não responder perguntas que incomodem você;

**SE EU TIVER DÚVIDAS SOBRE OS MEUS DIREITOS OU QUISER FAZER UMA RECLAMAÇÃO, COM QUEM EU FALO?**

Fale diretamente com o Comitê de Ética em Pesquisa do Hospital de Câncer de Barretos. Este comitê é formado por pessoas que analisam a parte ética dos estudos e autorizam ele acontecer ou não. Você pode entrar em contato com este Comitê. O horário de atendimento é de 2ª a 5ª feira, das 8h00 às 17h00, e 6ª feira, da 8h00 às 16h00. O horário de almoço é de 12h00 às 13h00.

**SE EU TIVER DÚVIDAS SOBRE O ESTUDO, COM QUEM EU FALO?**

Fale diretamente com o pesquisador responsável. As formas de contato estão abaixo:

**Hospital de Câncer de Barretos:**

Nome do pesquisador: Ricardo dos Reis / Gabriela da Silva Oliveira

Formas de contato: Ginecologia Oncológica de 2ª a 6ª das 8h00 às 17h00, horário de almoço é de 12h00 às 13h00.

**CAMPO DE ASSINATURAS**

|  |  |  |  |  |
| --- | --- | --- | --- | --- |
| Nome por extenso do participante de pesquisa ou do representante legal |  | Data |  | Assinatura |

|  |  |  |  |  |
| --- | --- | --- | --- | --- |
| Nome por extenso do pesquisador |  | Data |  | Assinatura |

|  |  |  |  |  |
| --- | --- | --- | --- | --- |
| Nome por extenso da testemunha imparcial (para casos de analfabetos, semi-analfabetos ou portadores de deficiência visual) |  | Data |  | Assinatura |
