# Supplementary material for: Emotional impact according to the way cancer patients are conducted to the surgical center: A randomized clinical trial comparing ambulation to the stretcher
Source: PLoS One. 2025 Apr 22;20(4):e0320856. doi: 10.1371/journal.pone.0320856 (PMC12013869; doi:10.1371/journal.pone.0320856)
Supplement: Attachment — Submitted filename: Response_to_Reviewers_auresp_2.docx [file pone.0320856.s007.docx]

**Answers to the Reviewers:**

Dear Dr. Alexandre Bonatto

Academic Editor

We sincerely appreciate the consideration of our manuscript PONE-D-24-33555R1 “Emotional impact according to the way cancer patients are conducted to the surgical center: a randomized clinical trial comparing ambulation to the stretcher” for publication in PLOS ONE. We are very happy to learn that the revised version was deemed suitable for publication, with the approval of the reviewers, and that the suggestions received were helpful in further improving the work. We would like to inform you that all of the reviewers' suggestions were carefully reviewed and implemented, in accordance with the received guidelines. Once again, we thank you for the opportunity and are available for any further requests or additional clarifications.

**Journal Requirements:**

**Comment 1.** Please review your reference list to ensure that it is complete and correct. If you have cited papers that have been retracted, please include the rationale for doing so in the manuscript text, or remove these references and replace them with relevant current references. Any changes to the reference list should be mentioned in the rebuttal letter that accompanies your revised manuscript. If you need to cite a retracted article, indicate the article’s retracted status in the References list and also include a citation and full reference for the retraction notice.

***Answer 1:*** *Thank you very much for your guidance. We have conducted a careful review of the reference list and, as suggested, replaced the study that had been retracted with the original publication of the HADS scale. This change was made to ensure the accuracy and relevance of the cited references. This change can be found on page 17, lines 423-424, reference nº 13.*

**Reviewers' comments:**

**Review Comments to the Author**

**Reviewer #4:** The authors have addressed my queries thoroughly and carefully. I have reviewed their responses and the amended version with tracked changes. I only have a few minor comments the authors may wish to consider:

**Comments 1:** The amended flowchart looks better, but the box “Did not follow randomisation” is still confusing as the numbers underneath do not make much sense (i.e. numbers do not add up). If those not following randomisation still took part, what about having both the numbers for those following and those not following randomisation? Similarly, this could also be specified in the final two boxes, both for the stretcher group and the ambulation group (this information was provided in the response to reviewers, but I could not find it in the amended figure). Consider using the CONSORT template as it accounts for these issues: <https://pmc.ncbi.nlm.nih.gov/articles/PMC2844943/>

***Answer 1:*** *We appreciate your comment and the detailed considerations. We have updated the flowchart to make it clearer and more informative. We changed the description from 'did not follow randomization' to 'crossover,' indicating that participants switched groups after randomization. Additionally, we included a caption in the flowchart explaining that this group change occurred due to unforeseen logistical issues related to hospital organization. Since the primary objective of the study was to assess anxiety levels based on the mode of transport, we decided to keep these patients in the study, even though they did not follow the initially assigned mode of transport. These changes can be found on page 7, lines 181-184, and in the document titled 'Fig-1,' attached to the submission platform.*

**Figure 1. The CONSORT flow diagram.**

This is the Figure 1 legend.

***Not eligible reasons**

- 5 patients were over 70 years of age.

- 8 patients required assistance to ambulate.

- 4 patients had cognitive impairment.

- 10 patients were using antidepressant/ anxiolytic medications.

- 18 patients were at moderate/high risk for falls**.**

**** Reasons for Losses**

All patients, from both groups, were referred to the surgical center to undergo the procedure before completing the questionnaires to assess anxiety and depression, and before signing the informed consent form.

*****Crossover**

Logistical challenges led to 30 participants deviating from randomization: 12 assigned to stretcher group walked instead, and 18 assigned to ambulation group were transported by stretcher. As the study focused on satisfaction, anxiety, and depression based on actual transport, analysis was conducted accordingly.

**Comment 2:** Text under “This is the Figure 1 legend” (line 173, page 7): eligible instead of elegible? Please note that the figure may also need to be edited.

***Answer 2:*** *Thank you very much for your observation. We have made the correction to the word as pointed out. This change can be found on page 7, line 170, and in the document titled 'Fig-1,' attached to the submission platform.*

**Comment 3:** Legends under all tables: what is meant by “ignored values”? Is this the same as missing data?

***Answer 3:*** *We appreciate your comment and question. 'Ignored values' refers to missing data. To avoid any possible confusion, we have changed all the captions to 'missing data.' These changes can be found in lines: 188, 217, and 236.*

**Comment 4:** Discussion, page 15, line 336: instead of “with all types of surgical procedures” consider saying “undergoing all types of surgical procedures” or something along these lines.

***Answer 4:*** *Thank you for your comments and suggestion. We have made the change as suggested. This change can be found in lines 336-337.*

**Comment 5:** Discussion, page 15, line 347: Based on the study findings only, or based on the study findings and other evidence from the literature?

***Answer 5:*** *Thank you for your comments and suggestion. We have modified the sentence to make it clearer as 'Based on the study findings and other evidence from the literature.' This information can be found in lines 346-347.*

| *Ricardo dos Reis* |  | *Gabriela da Silva Oliveira* |
| --- | --- | --- |
